# Stage I and II Small-Cell Lung Cancer—New Challenge for Surgery

**DOI:** 10.1007/s00408-022-00549-8

**Published:** 2022-06-30

**Authors:** Fabian Doerr, Sebastian Stange, Maximilian Michel, Georg Schlachtenberger, Hruy Menghesha, Thorsten Wahlers, Khosro Hekmat, Matthias B. Heldwein

**Affiliations:** 1 Department of Cardiothoracic Surgery, University Hospital of Cologne, University of Cologne, Kerpener Straße 62, 50931 Cologne, Germany; 2 Department of Thoracic Surgery, Regiomed-Klinikum Coburg GmbH, Coburg, Germany; 3 Institute of Zoology, Faculty of Mathematics and Natural Sciences, University of Cologne, Cologne, Germany

**Keywords:** Small-cell lung cancer, Surgery, Meta-analysis, Mean-survival

## Abstract

**Purpose:**

The recommended treatment for small-cell lung cancer (SCLC) currently is surgery in stage I disease. We wondered about stage II SCLC and present a meta-analysis on mean-survival of patients that underwent surgery for stage I and II compared to controls.

**Methods:**

A systematic literature search was performed on December 01st 2021 in Medline, Embase and Cochrane Library. We considered studies published on the effect of surgery in SCLC since 2004 and assessed them using ROBINS-I. We preformed I^2^-tests, Q-statistics, DerSimonian-Laird tests and Egger-regression. The meta-analysis was conducted according to PRISMA.

**Results:**

Out of 6826 records, seven studies with a total of 11,241 patients (‘surgery group’: 3911 patients; ‘non-surgery group’: 7330; treatment period: 1984–2015) were included. Heterogeneity between the studies was revealed in absence of any publication bias. Patient characteristics did not differ between the groups (p-value > 0.05). The mean-survival in an analysis of patients in stage I was 36.7 ± 10.8 months for the ‘surgery group’ and 20.3 ± 5.7 months for the ‘non-surgery group’ (p-value = 0.0084). A combined analysis of patients in stage I and II revealed a mean-survival of 32.0 ± 16.7 months for the ‘surgery group’ and 19.1 ± 6.1 months for the ‘non-surgery group’ (p-value = 0.0391). In a separate analysis of stage II, we were able to demonstrate a significant survival benefit after surgery (21.4 ± 3.6 versus 16.2 ± 3.9 months; p-value = 0.0493).

**Conclusion:**

Our meta-analysis shows a significant survival benefit after surgery not only in the recommended stage I but also in stage II SCLC. Our data suggests that both stages should be considered for surgery of early SCLC.

## Introduction

Lung cancer is the most common type of malignant neoplasm with an incidence of approximately two million worldwide [1]. While small-cell lung cancer (SCLC) accounts for only 15% of all malignant lung-tumours, it is the fifth leading cause of cancer death [2]. SCLC is characterized by high-grade malignancy with rapid growth of the primary lesion and early spreading to mediastinal lymph nodes or distant organs [3]. The mean age at the time of SCLC diagnosis is approximately 65 years with tobacco smoke being the main risk factor [2].

Current guidelines recommend curative treatment in stage I to III [4, 5]. Treatment of SCLC is multimodal and typically consists of chemotherapy, radiation and in selected cases a surgical approach [4, 5]. For patients presenting with an extensive disease stage (M1), guidelines suggest palliative rather than curative care [4, 5].

Surgery is currently only recommended for stage I disease (Grade 2C; American College of Chest Physicians (ACCP)), which is diagnosed in approximately 5% of all SCLC cases. [4]. However, a substantially larger fraction of patients (approximately 60%) presents with stage II and III disease and is thus considered unresectable. For these cases, chemotherapy with concurrent radiotherapy is the suggested option [4, 5]. Despite promising early responses, most patients in these stages relapse. Therefore, surgery to treat SCLC has gained momentum over the past decade not only in stage I disease but also in stage II. Nonetheless, resection as a curative approach beyond stage I remains controversial [6].

The aim of this meta-analysis was to assess the role of surgery in the curative treatment of stage I (T1-T2aN0) and II (T2b-T3N0 or T1-T2N1) SCLC. To achieve this, mean-survival rates were compared between patients who underwent surgery and patients who did not.

## Material and Methods

### Study Inclusion and Exclusion Criteria

**Fig. 1:**
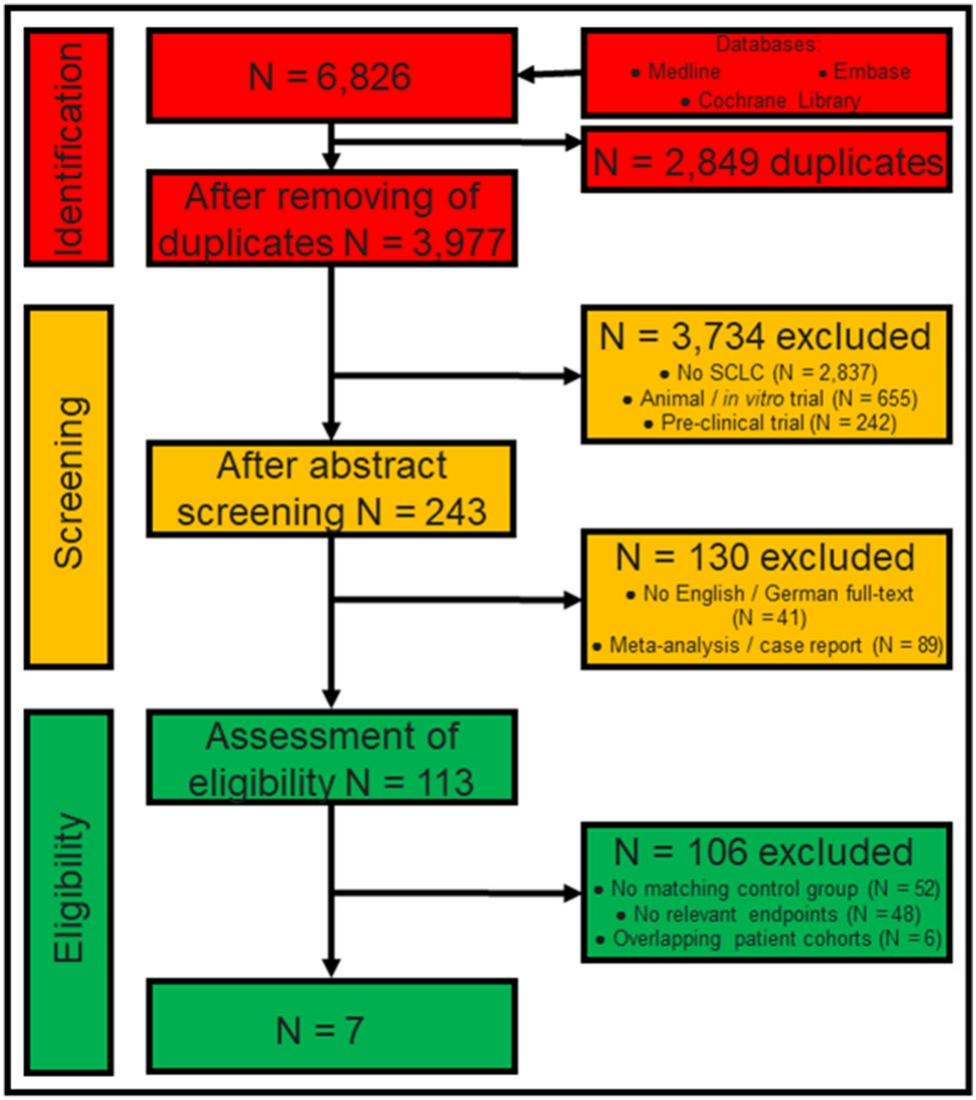
Title: Flow-chart of literature research. The figure displays ‘identification’ (red) of studies after literature research in three databases. a ‘screening’ (orange) procedure followed to identify the relevant articles which are finally ‘eligible’ (green) for inclusion into the meta-analysis. Colored boxes in the middle display number of articles at each step of assessment. Colored boxes in the right display the number of excluded articles and the reason of exclusion. SCLC: Small-cell lung cancer

This unregistered systematic review was performed in accordance with the Preferred Reporting Items for Systematic Reviews and Meta-Analyses (PRISMA) guidelines [7]. As this work is a meta-analysis an ethics committee approval was not considered. Databases were queried for randomised prospective trials and retrospective studies investigating the effect of surgery in stage I and II SCLC. We only considered work published since 2004. Since staging of lung cancer has changed over the past decades, modern staging tools such as a Computed tomography (CT-scan) were considered mandatory upon study inclusion [8]. An exclusive limitation to staging with Positron Emission Tomography scan (PET-CT) and Magnetic Resonance Imaging (MRI) of the brain was not executed. Consequently, patient recruitment in the original study had to be within the last 35 years to avoid bias due to inaccurate staging. We identified and analysed studies in accordance with the criteria listed in Fig. 1.

The ‘non-surgery group’ was defined as radio-/chemotherapy treatment only, with a majority of patients being treated with a combination of both. All patients who underwent surgical treatment alone or in any combination with neoadjuvant or adjuvant therapy were defined as ‘surgery group’. The definitions of clinical end-points were taken from the primary publications.

### Search Strategy

Two authors (FD and SS) performed an independent literature search on December 01^st^ 2021 in the Medline, Embase and the Cochrane Library databases. We restricted the languages to English and German. A reference management software (Endnote, Version X9.2, Clarivate Analytics, Spring Garden, Philadelphia, United States) was used to organize all relevant articles. An initial selection was performed by reviewing all titles and abstracts. Full-text was recovered and reference-lists of these papers were further screened to identify other publications fulfilling the above criteria. In case of several publications per patient collective, the study with the most complete dataset was selected for our analysis to avoid inclusion of patients multiple times. Figure 1 provides a detailed flow-chart of the search strategy.

### Data Extraction and Quality Assessment

All relevant data including demographic data and end-points of interest were extracted from the original studies. The first or the senior author of an original study was contacted in case of missing information. Study quality and risk of bias were assessed by two independent investigators (FD and SS) using ROBINS-I criteria [9].

### Statistical Analysis

Statistical analysis was performed using the StatsDirect software package (Version 3.2.10, StatsDirect Ltd, Birkenhead, Merseyside, United Kingdom). Throughout our statistical analysis, a p-value < 0.05 was considered significant. We compared treatment groups of each original study with a log-rank test. The hazard ratio (HR) and the 95% confidence interval (95%-CI) of each data set was calculated. The pooled HR of all studies was analysed and expressed as a Forest plot. Q-statistics (p-value < 0.05) and I^2^-tests (I^2^ > 50%) were performed to evaluate heterogeneity between studies [10]. In the presence of clinical and statistical heterogeneity, the DerSimonian and Laird random-effects model was implemented [11]. The pooled treatment effect estimate was calculated as a weighted average of the treatment effects so that an HR < 1 favoured the ‘surgery group’ over the control group. Here, the size of squares in the plot displays the sample size. Publication bias was assessed by Egger’s weighted regression statistic with a p-value < 0.05 indicating significant publication bias among included studies.

## Results

### Literature Search

**Fig. 2:**
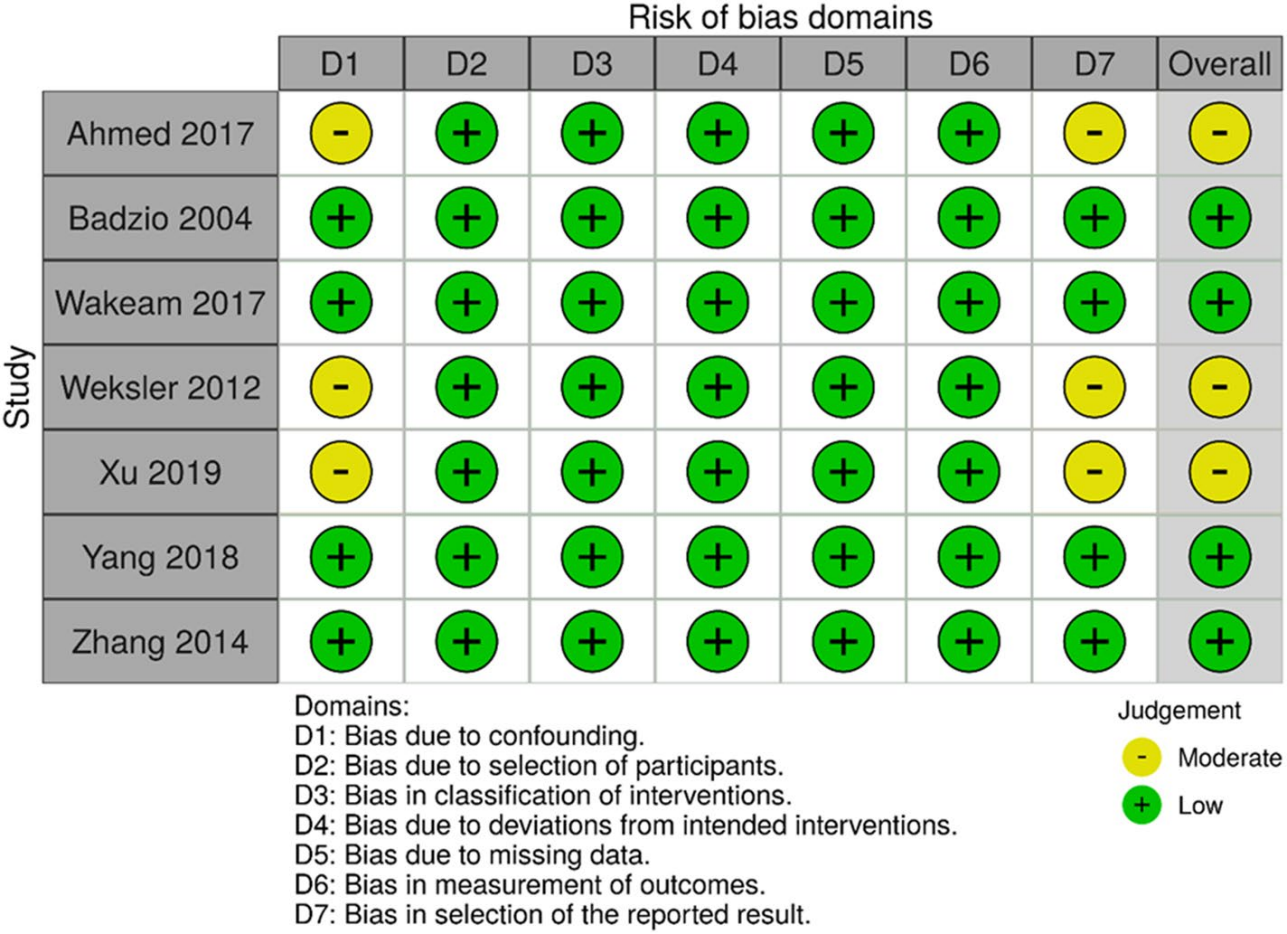
Title: Overall risk of bias according to ROBINS-I

From an initial set of 6826 papers found in the systematic literature research, seven studies were included in the meta-analysis (Fig. 1). According to ROBINS-I the overall risk of bias in the studies included was low or moderate (Fig. 2). Publication dates ranged between 2004 and 2019 (Table 1). The longest period of patient recruitment was 20 years (1988–2007) performed by Weksler et al. [12]. The most recent paper by Xu et al. had the shortest recruitment period of 6 years (2010–2015) [13]. All studies are retrospective. Five studies are based on national data registries and two studies compile single centre data. Two studies used a pair-match analysis [14, 15] (Table 1).

**Table 1:** Overview of all original studies

Author	Year	Period	Origin	Mean-surv. (months)		Patients (N)		
				Surg	NS	All	Surg	NS
Stage I								
Ahmed	2017	2007–2013	SEER	53.0	27.0	1358	543	815
Badzio	2004	1984–1996	SC*	28.0	13.0	52	27	25
Wakeam	2017	2004–2013	NCDB*	38.6	22.9	2620	1310	1310
Weksler	2012	1988–2007	SEER	38.0	16.0	2686	683	2003
Yang	2018	2003–2011	NCDB**	54.9	25.9	2301	681	1620
Zhang	2014	1995–2013	SC	25.8	22.5	20	11	9
Stage II								
Badzio	2004	1984–1996	SC*	17.0	12.0	43	21	22
Wakeam	2017	2004–2013	NCDB*	23.4	20.7	670	335	335
Weksler	2012	1988–2007	SEER	25.0	14.0	880	212	668
Xu	2019	2010–2015	SEER	20.0	18.0	599	83	516
Zhang	2014	1995–2013	SC	11.5	17.4	12	5	7
Jin	2018	2004–2013	SEER**	34.0	24.0	1186	154	1032
Peng	2019	2004–2015	SEER**	26.0	15.0	2453	687	1766
Schreiber	2010	1988–2002	SEER	65.0	15.0	2226	231	1995
Uprety	2019	2004–2013	NCDB	61.7	31.2	1026	486	540
Varlotto	2011	1988–2005	SEER	50.0	20.0	1053	361	692
Wang	2020	2004–2014	SEER**	35.0	19.9	2246	618	1628

Summary of each original study including year of publication, period of patient recruitment, data origin, a comment on details of each original study including stage analysed mean-survival in months, and number of patients in each treatment group

Studies in the lower part of the table, displayed in *italic* were excluded from this meta-analysis due to overlapping patient cohorts from similar data sources

*Mean-Surv.* Mean-survival, *NCDB* National Cancer Database, *NS* Non-surgery group, *SC* Single centre, *SEER* Surveillance, Epidemiology, and End Results database, *Surg.* Surgery group, *Pair-match analysis, **stage I and II combined

Despite meeting the inclusion criteria, we excluded six studies from this meta-analysis due to overlapping patient cohorts or similar data sources [16–21]. Data for these six excluded studies is displayed in the lower section of Table 1.

### Patient Details

The seven included studies summed a total of 11,241 patients. Of these, 3911 patients are in the ‘surgery group’ and 7330 in the ‘non-surgery group’. Patients’ mean age was 64.2 ± 5.9 years, and 55.3 ± 15.4% of all patients were male. These patient characteristics did not significantly (p-value > 0.05) differ between the ‘surgery’ and ‘non-surgery group’ (Table 2). Further stage-specific analysis on both groups including the level of fitness according to the ‘Charlson/Deyo comorbidity condition’ score (CDCC) are summarized in Table 3.

**Table 2:** Summarized baseline characteristics

Number patients	Mean age (years)	p-value	Male (%)	p-value
All patients (11,241)	64.2 ± 5.9		55.3 ± 15.4	
Surg. group (3911)	62.7 ± 5.4	0.37	55.6 ± 17.3	0.49
Non-surg. group (7330)	65.9 ± 7.1		54.9 ± 13.9	

Summary of patients baseline characteristics including number of patients in each group, mean age in years, and gender distribution in male %. *Non-surg*. Non-surgery group, *Surg.* Surgery group

**Table 3:** Detailed baseline characteristics

	Surgery group	Non-surgery group	P-value	Stage
Age (years) [range / SD]				
Ahmed	68 [61–73]	70 [63–77]	0.13	I
Wakeam	68 [62–74]	69 [62–75]	0.23	I
Yang	65.8 [± 8.3]	65.7 [± 9.9]	0.73	I
Badzio	57 [29–70]	54 [36–71]	**0.03**	I + II
Weksler	67.8 [± 8.9]	68.9 [± 10.1]	**0.01**	I + II
Zhang	57 [32–75]	56 [23–84]	0.64	I + II
Wakeam	67 [59–74]	67 [60–74]	0.54	II
Xu	66.6 [± 8.5]	68.3 [± 9.4]	0.07	II
Male (%)				
Ahmed	51.0	45.3	0.26	I
Wakeam	43.5	43.7	0.66	I
Yang	42.9	43.8	0.68	I
Badzio	85.0	78.0	0.27	I + II
Weksler	48.2	48.8	0.76	I + II
Zhang	76.0	71.8	0.38	I + II
Wakeam	49.9	49.9	1.0	II
Xu	38.6	41.5	0.63	II
Race, white (%)				
Ahmed	85.0	92.8	**0.02**	I
Wakeam	91.1	91.9	0.75	I
Yang	92.4	89.9	**0.01**	I
Weksler	90.5	85.9	**0.01**	I + II
Wakeam	90.7	92.2	0.68	II
Xu	89.2	85.1	0.45	II
CDCC score 0				
Yang	44.8	64.9	**0.01**	I
Wakeam	49.0	50.8	0.65	I
Badzio	60.0	58.0	0.57	I + II
Wakeam	49.6	46.6	0.59	II
CDCC score 1				
Yang	39.8	24.7	**0.01**	I
Wakeam	36.2	35.2	0.65	I
Badzio	36.0	33.0	0.57	I + II
Wakeam	37.9	41.8	0.59	II
CDCC score 2 +				
Yang	15.4	10.4	**0.01**	I
Wakeam	14.8	14.1	0.65	I
Badzio	4.0	9.0	0.57	I + II
Wakeam	12.5	11.6	0.59	II

Bold values denote statistical significance at the p < 0.05

*CDCC* Charlson/Deyo comorbidity condition, *SD* standard deviation

All patients in the ‘surgery group’ and in the ‘non-surgery group’ received chemotherapy. The only exceptions to this are the studies by Wakeam et al. [15] and Xu et al. [13], in which only 71% (stage I) and 80% (stage II) of the resected patients were treated with chemotherapy, respectively. Furthermore, it must be mentioned that chemotherapy data are not available on the SEER database.

The proportion of patients who received a combination of chemotherapy and radiation therapy in the ‘non-surgery group’ ranged between 50% in the study by Xu et al. and 100% in the study by Yang et al. [13]. Nonetheless, 75% of all patients in the ‘non-surgery group’ were treated with a combined radiochemotherapy.

### Mean-Survival Analysis in Stage I

**Fig. 3:**
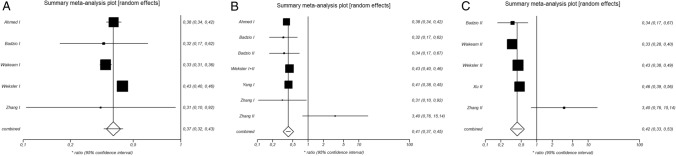
Title: Summary meta-analysis plot in stage I (**A**), in stage I and II combined (**B**), and in stage II isolated (**C**). The figure displays the results of the meta-analysis in stage I (sub-figure **A**), combined stage I and II (sub-figure **B**), and separate stage II (sub-figure **C**). Names on the left stand for first author of original study. Studies were mentioned multiple times in case different SCLC stages were included in one analysis. Hazard ratio < 1 provides evidence for superiority of surgery. Size of squares displays sample size. Numbers on the right display hazard ratio and 95%-confidence interval for each study

The Q-statistic for the mean-survival endpoint was significantly different (p-value = 0.0013) and the I^2^-test showed 77.6% inconsistency (95%-CI: 23.6–88.9%). This provides evidence for significant statistical heterogeneity between included studies. We therefore implemented the DerSimonian and Laird random-effects model. The pooled hazard ratio was 0.4 (95%-CI: 0.32–0.43) and the Z-test was −13.6 (p-value < 0.0001). This suggests that the ‘surgery group’ showed significant improvement in the mean-survival endpoint compared to control patients (Fig. 3A). Egger’s weighted regression statistic signified that there was no publication bias (p-value = 0.698). Mean-survival was 36.7 ± 10.8 months in the ‘surgery group’ versus 20.3 ± 5.7 months in the ‘non-surgery group’. Therefore, surgical intervention improved mean-survival significantly (p-value = 0.0084) (Fig. 3A).

### Mean-Survival Analysis in Stage I and II Combined

Since some groups consider surgical treatment for patients in stage II SCLC, it is plausible to include this sub-population in an analysis together with stage I patients. The Q-statistic for the mean-survival endpoint was significant (p-value = 0.046) and the I^2^-test suggested 53.3% inconsistency (95%-CI: 0–78.2%), again showing significant heterogeneity between included studies. We applied the DerSimonian and Laird random-effects model as in the other analysis. The pooled hazard ratio of 0.4 (95%-CI: 0.37–0.45) and the Z-test was −17.9 (p-value < 0.0001). These results suggest a significant survival benefit for patients in stage I and II after surgery (Fig. 3B). Egger’s weighted regression statistic showed no significant publication bias (p-value = 0.925). Consequently, surgical intervention improved mean-survival in stage I and II SCLC (p-value = 0.0391). Mean-survival was 32.0 ± 16.7 months in the ‘surgery group’ versus 19.1 ± 6.1 months in the ‘non-surgery group’ (Fig. 3B).

### Mean-Survival Analysis in Isolated Stage II

In order to show that the survival advantage in the combined analysis of both stages is not exclusively due to the good results of stage I, we analysed stage II separately. Here the Q-statistic for the mean-survival endpoint was significant (p-value = 0.0035) and the I^2^-test suggested 74.4% inconsistency (95%-CI: 3.0–87.7%), again showing significant heterogeneity between included studies. We applied the DerSimonian and Laird random-effects model as in the other analysis. The pooled hazard ratio of 0.4 (95%-CI: 0.33–0.53) and the Z-test was −7.5 (p-value < 0.0001). These results suggest a significant survival benefit for patients in stage II after surgery (Fig. 3C). Egger’s weighted regression statistic showed no significant publication bias (p-value = 0.51). Consequently, surgical intervention improved mean-survival in stage II SCLC (p-value = 0.0493). Mean-survival was 21.4 ± 3.6 months in the ‘surgery group’ versus 16.2 ± 3.9 months in the ‘non-surgery group’ (Fig. 3C).

## Discussion

### Guideline-Concordant Use of Surgery in T1/2N0 Situation

The ACCP guidelines give a grade 2C recommendation for surgical therapy in SCLC stage I. In this stage, surgery is preferred over any non-surgical therapy [4]. The National Comprehensive Cancer Network (NCCN) [5] and the American Society of Clinical Oncology (ASCO) [22] recommend resection as initial treatment for node-negative SCLC patients in stage I after pathologic mediastinal staging. The European Society for Medical Oncology (ESMO) recommends surgery for patients in T1/2, N0/1 situation without mediastinal involvement. Surgical therapy should be followed by chemotherapy according to all guidelines.

### Are Stage I and Stage II SCLC Ready for Surgery?

Several authors, among them Ahmed et al. who recently analysed the SEER database [23], support our opinion that survival is improved by surgery in stage I SCLC. The mean-survival in our meta-analysis was 36.7 months in the ‘surgery group’ versus 20.3 months in the ‘non-surgery group’. Even if stage II is analysed separately, SCLC patients benefit significantly from resection and non-surgical treatment leads to a shortening of survival-time (21.4 versus 16.2 months). At this point, we would like to point out that some patients that were included in this meta-analysis were potentially not staged with PET-CT and MRI of the brain.

It is not to be expected that the results of our meta-analysis would significantly change as a result of including the six studies that were excluded due to overlapping patient cohorts from similar data sources. Uprety et al. report a 30 months survival benefit for surgical patients in stage I of the NCDB database [19]. The SEER database was sourced by several authors. Schreiber et al. and Varlotto et al. report on stage I patients from the SEER database between the years 1988 to 2005. In these studies, the authors showed a survival benefit of 50 and 30 months, respectively [18, 20]. Three studies (Jin et al., Peng et al., and Wang et al.) analysed mean-survival of stage I and stage II SCLC patients from the SEER database between 2004 and 2015. The mean-survival benefit in these studies ranged from 10 to 15 months [16, 17, 21]. Furthermore, Zhu et al. published a single centre study in 2013 and reported a significant mean-survival benefit for surgically treated patients in stage I and II compared to patients treated with chemoradiotherapy (91.0 months versus 34.6 months; p-value: 0.004) [24]. This study could not be included into our meta-analysis since some relevant data were not reported by the authors.

Our meta-analysis data provide evidence for a significant mean-survival benefit after surgery in early-stage SCLC. This finding might suggest expanding the role of surgery to stage II SCLC, for which there is currently no clear guideline recommendation for resection. We believe that stage I and stage II SCLC are ready for surgery.

### No Improvement on Prognosis of SCLC Since Decades

Unlike NSCLC, in which the treatment options have been revolutionized in recent years and the prognosis has been significantly improved even for advanced stages, the overall 5-year survival rates in SCLC remain below 10% [25]. But there is a glimpse of hope. Today, with a better understanding of the SCLCs biology, we have numerous therapeutic options, not least targeted immunotherapies [26]. Recently we showed that SCLC displays an actionable dependence on ATR/CHK1-mediated cell cycle checkpoints [27]. Nevertheless, potential cellular and molecular mechanisms need to be further investigated. We share the opinion of Byers et al. that translational SCLC research is severely affected by the limited access to human tumour tissue [25].

### Resection Rates Need to Increase, But How Radical Do We Have to Operate?

In accordance with the guidelines, surgery should be preferred to non-surgical therapy in stage I as it improves long-term prognosis [4, 5]. However, the number of operations stagnated in the past decade [23] and has reached only 10% in potentially resectable patients [6]. Today, less than a third of all stage I patients are evaluated for surgery [6, 15, 23]. It follows that surgery is significantly underused in SCLC [28].

The data of Weksler et al. indicate that wedge resection results in significantly worsened median survival compared to lobectomy or pneumonectomy (39 months versus 28 months, p-value < 0.001) [12]. According to Schreiber et al. the median survival-time was longest after lobectomy, followed by sublobar resection, pneumonectomy and lack of surgery (40 months, 23 months, 20 months and 13 months, respectively) [18]. In 2018 Che et al. reported a median survival of 34 months after lobectomy. Any type of sublobar resection resulted in a shorter median survival of 17 months [29]. In this context, it is interesting that Varlotto et al. provide evidence of optimal local control after lobectomy which leads to superior survival. The authors suggest inferior outcome after sublobar resection compared to lobectomy, but they report a survival advantage of both surgical techniques compared to radiotherapy alone [20].

This section shows the importance of surgery in SCLC, especially with a focus on lobectomy. In attempt to avoid a pneumonectomy it is not uncommon to perform a sleeve resection in SCLC due to centrally growing tumours [30]. Since it is important to assess the need of postoperative radiation of the mediastinum a systematic lymph-node dissection should always be performed beside lobectomy [31].

### Is the Impact of Surgery on Long-Term Survival Really That Great?

The survival benefit of surgery in stage I and stage II SCLC appears impressive in this meta-analysis. Could there be an underestimated bias of patient selection? It is possible that it is not the surgery itself, but rather the selection factors enabling surgery, that play a critical role in favourable outcomes. Hence, it is debatable, whether patient selection or surgery lead to favourable outcomes.

The subset of SCLC patients eligible for surgery is characterized by early clinical stage with less tumour burden, good physical performance and lack of significant comorbidities [20, 28]. Consequently, these patients have a better prognosis irrespective of whether surgical treatment is attempted or not. This favourable preselection might influence the results of the published studies and distort the efficacy of surgery in SCLC [6, 14]. We addressed this potential bias due to preselection in our meta-analysis by evaluating performance scores such as ECOG (Eastern Cooperative Oncology Group) to assess the fitness of patients in both groups whenever possible. Nevertheless, not all original studies performed a pair-matching in regard of the patients’ fitness. Since such scores were not reported regularly, patients that underwent surgery might have had a better health status at time of intervention than their non-surgical counterparts. Consequently, a bias due to confounding might exist in selected cases.

### The Pre 90 s RCTs Are Still the Spoilsport for Surgery Today

Current surgical guidelines in SCLC are largely based on three RCTs by Fox et al. [32], Lad et al. [33] and Liao et al. [34], which undoubtedly guarantee the best level of evidence [35], and which show little to no improvement of surgery over alternate treatment options. A recent Cochrane review pointed out difficulties in these studies’ interpretation under today’s standards [36]. We believe that evaluating the role of surgery in SCLC based on these three RCTs seems not justified today due to several reasons. First, the patients’ recruitment period already started in 1962 [32]. Second, the total number of patients is small and the staging procedures led to inclusion of participants in advanced SCLC stages, which would not be suitable for surgery today. Third, the treatment in these RCTs is heterogenous and does not fulfil today’s surgical standards and recommendations especially in regard of the high number of pneumonectomies’ and explorative thoracotomies. Lastly, results after surgery are biased due to a high percentage of incomplete or not executed resections. Despite all, Lad et al. and Liao et al. do not report a significant inferiority of surgical treatment [33, 34].

## Conclusion

We provide a meta-analysis on stage I and stage II SCLC patients that reveals superior long-term outcome after surgery. Patients in both stages gain significant lifetime through resection. Based on data of our meta-analysis, we believe that SCLC patients in stage I and stage II should be considered for surgery. All analysed data in this systematic review are of a retrospective nature. Today this might still be the top of the pyramid of evidence-based medicine. Nevertheless, we want to urgently call for a high quality and high volume multi-institutional randomized control trial on the role of surgery in SCLC, which clearly has the potential to change future guidelines.
